# Blood RNA expression profiles undergo major changes during the seventh decade

**DOI:** 10.18632/oncotarget.12098

**Published:** 2016-09-17

**Authors:** Marius Gheorghe, Claudia Schurmann, Marjolein J. Peters, André G. Uitterlinden, Albert Hofman, Reiner Biffar, Georg Homuth, Uwe Völker, Joyce BJ van Meurs, Vered Raz

**Affiliations:** ^1^ Department of Medical Informatics, Erasmus University Medical Center, Rotterdam, 3000 CA, The Netherlands; ^2^ Department of Internal Medicine, Erasmus University Medical Center, Rotterdam, 3000 CA, The Netherlands; ^3^ Department of Functional Genomics, Interfaculty Institute for Genetics and Functional Genomics, University Medicine Greifswald, Greifswald, 17475 DE, Germany; ^4^ Department of Human and Clinical Genetics, Leiden University Medical Centre, Leiden, 2300 RC, The Netherlands; ^5^ Department of Epidemiology, Erasmus University Medical Center, Rotterdam, 3000 CA, The Netherlands; ^6^ The Charles Bronfman Institute for Personalized Medicine, Genetics of Obesity and Related Metabolic Traits Program, Icahn School of Medicine at Mount Sinai, New York, NY 10029, United States of America; ^7^ Department of Prosthodontics, Gerodontology and Biomaterials, University Medicine Greifswald, Greifswald, 17475 DE, Germany

**Keywords:** blood aging, population based studies, RNA expression profiles, sample weighting, b-spline regression model

## Abstract

Genome-wide alterations in RNA expression profiles are age-associated. Yet the rate and temporal pattern of those alterations are poorly understood. We investigated temporal changes in RNA expression profiles in blood from population-based studies using a quadratic regression model. Comparative analysis between two independent studies was carried out after sample-weighting that downsized differences in sample distribution over age between the datasets. We show that age-associated expression profiles are clustered into two major inclinations and transcriptional alternations occur predominantly from the seventh decade onwards. The age-associated genes in blood are enriched in functional groups of the translational machinery and the immune system. The results are highly consistent between the two population-based studies indicating that our analysis overcomes potential confounders in population-based studies. We suggest that the critical age when major transcriptional alterations occur could help understanding aging and disease risk during adulthood.

## INTRODUCTION

Age-dependent tissue deterioration characterizes physiological aging in multi-cellular organisms. Impaired maintenance of cellular homeostasis contributes to aging, partly caused by genome-wide transcriptional alterations affecting multiple gene networks [1]. In humans, the aging-associated gene network pattern is highly complex. Compared with model organisms, in humans the adulthood phase is much longer. Thus, slow progression in tissue and molecular alterations can contribute to the complexity of the aging process.

Most often, age-associated physiological and molecular alterations are extracted using linear regression models (few examples in: [2–7]). Linear regression assumes a constant change over time and therefore might be appropriate for organisms that aged over a short period [2]. In humans, however, adulthood spans from 50 to 80 years. It is very unlikely that the rate of age-associated changes progresses at a constant rate. The fitness of different regression models to describe age-associated physiological features demonstrated that a quadratic or a parabolic regression model are most suitable to describe age-associated changes [8]. We also reported that the fitness of a quadratic regression model to describe age-associated changes in expression profiles is higher than linear or cubic regression models [9]. Quadratic models have fewer assumptions compared with a linear model. Moreover, a quadratic model could be employed to identify the age when major changes occur (named here age-position). Using cross-sectional transcriptome studies, it was suggested that most transcriptional alterations in brain frontal cortex occur around the age of 42 [10], and in Vastus lateralis muscle major changes occur already in the fourth decade [11]. In both studies, two linear regression models were applied to identify the age-positions. Applying a quadratic regression model we indeed confirmed that major expression profiles are changed first in the fourth decade in both brain frontal cortex and Vastus lateralis muscle [9].

Ideally, the pattern of aging-associated molecular changes could be extracted from population-based datasets [12]. These datasets are cross-sectional, covering a broad age-range, and all subjects are included. Most population-based datasets are skewed in the old age, making a linear regression model unfit. Here we investigated age-associated molecular changes in whole blood from two population datasets. The Rotterdam Study (RS) cohort III [13] and the SHIP-TREND cohort [14, 15] were independently generated using RNA microarrays. After correcting for the skewed sample distribution across age, we demonstrate that an age-associated pattern of molecular changes is highly similar between the two datasets. We show that in whole blood major molecular changes occur only at the seventh decade, predominantly affecting the translation and immune cellular machineries.

## RESULTS

Dataset demographics of the RS and the SHIP-TREND are presented in Table 1. Each datasets was analyzed independently and subsequently compared for replication, confirmation and identification of the most consistent age-dysregulated genes. In both studies, all subjects are Caucasian. Gender distribution across age was similar and not skewed in both datasets (Supplementary Table S1). Outliers were not identified by using principal component analysis (Supplementary Figure S1). Therefore, all subjects were included and the models were not corrected for potential confounders. However, the sample distribution over age and the age-range differed between the two studies (Table 1 and Supplementary Figure S2), and a positive skewness was found in the RS dataset, indicating underrepresentation of elderly subjects (Supplementary Figure S2). To compensate for the uneven sample distribution we included a sample-weighting step, which downsized the effect of overrepresentation and underrepresentation in the population. Sample weighting was achieved using moving decade age groups inversely proportional to the size of their neighbourhood. We compared the number of significantly dysregulated probes (*p-value* < 0.05; false discovery rate (FDR)) between the non-weighted and weighted datasets and found that the percentage of overlapping age-associated genes between the weighted and non-weighted datasets was high for both the RS and SHIP-TREND dataset (76.7% for RS, 94.4% for SHIP-TREND; Table S2). Higher percentage of overlapping genes in the SHIP-TREND dataset is expected due to a more even distribution with age as compared to the RS dataset (Supplementary Figure S2). A higher number of genes were identified as age-associated after sample weighting compared with the non-weighted, in both datasets (Supplementary Table S2).

**Table 1:** Study demographics

	Samples	Age range	#Probes	#Annotated probes
**RS**	762	46–89	21238	15216
**SHIP-TREND**	991	21–81	48803	24928
Overlap (%)			19750 (93%)	13741 (90%)

Table shows the number of included study participants, age range in years, number of probes and number of annotated probes (using Entrez gene ID) for the RS and the SHIP-TREND dataset. The overlap between RS and SHIP-TREND is calculated out of the RS dataset.

The age-associated probes were identified using a basis spline (b-spline) quadratic regression model. To reduce assumptions in the model, the b-spline excluded control knots [9]. Significant probes were considered with a *p-value* < 0.05 (FDR). Out of all probes, 73% and 41.7% were significantly age-associated in the RS and in the SHIP-TREND dataset, respectively, however most significant probes (93.3% and 95.4%; (Supplementary Table S2)) had a fold change (FC) < 1.2 in absolute value. As fold change is crucial to assess the rate of changes over age, we applied a FC filter. We selected a FC ≥ 1.2 in absolute value as a threshold, based on a recent study suggesting that this numeric FC value renders more reproducible results [16].

**Figure 1:**
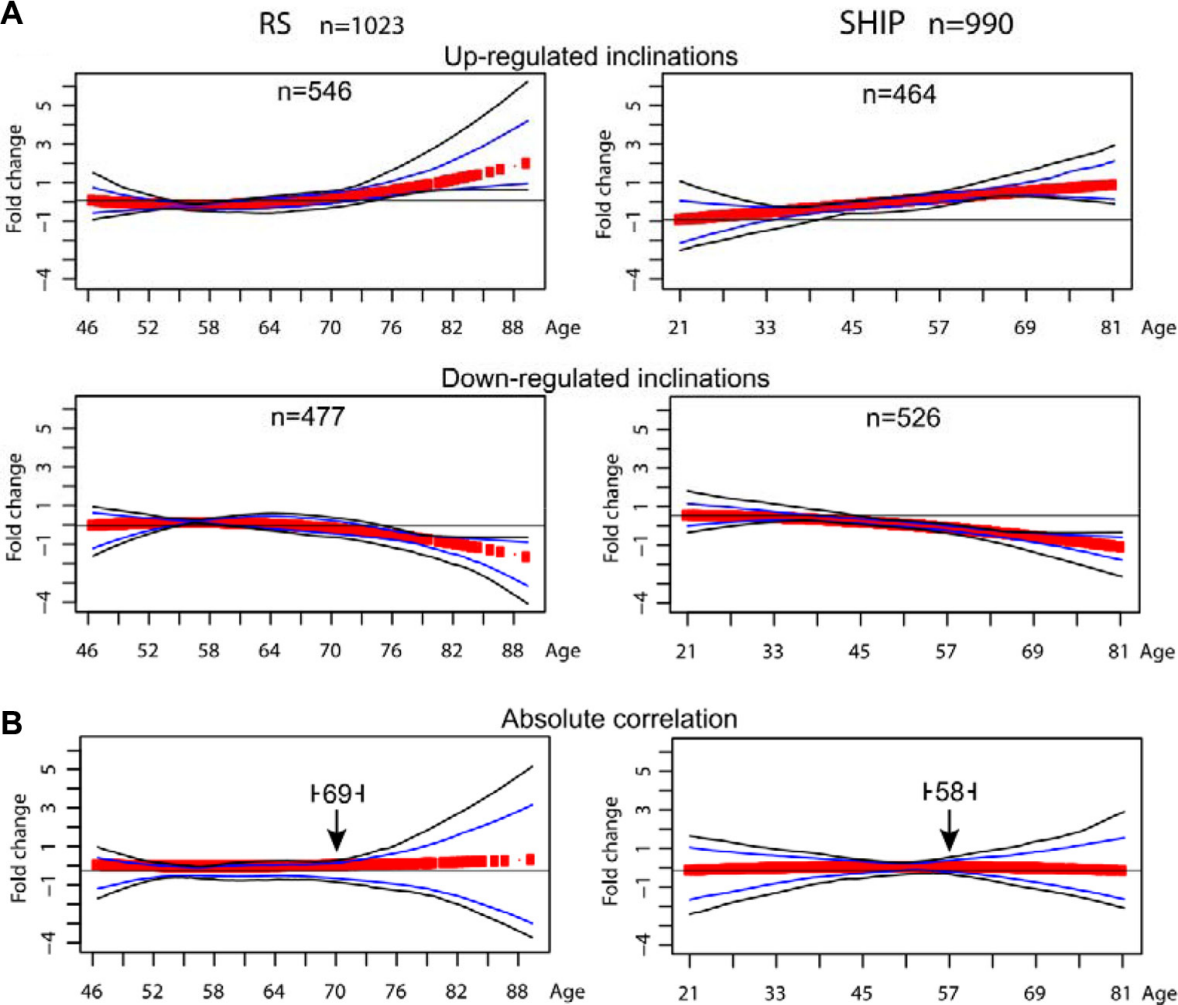
Trends of the clustered age-associated significant probes Scatter plots showing the trends of the mRNA expression profiles of the age-associated significant probes clustered using *k*-means algorithm. Clustering includes only probes with FDR < 0.05 and FC ≥ 1.2 in absolute value. Panel (**A**) shows the clusters identified using Euclidean distance as metric in the clustering algorithm, in the RS dataset (left) and in the SHIP-TREND dataset (right). The number of probes per cluster is depicted on top of every plot. Panel (**B**) shows the results of the *k*-means clustering using absolute correlation as distance metric. This facilitates the grouping of the probes presenting a symmetric expression profile. The arrowhead indicates the identified age-position in the RS dataset (left) and in the SHIP-TREND dataset (right).

The probes that passed the FDR < 5% and absolute FC ≥ 1.2 filtering criteria, 1023 in the RS and 990 in SHIP-TREND (Table 2) were clustered using *K*-means clustering method. *K*-means with Euclidean distance as a distance measure was used to identify the age-associated trends in the expression profile datasets, with the age being used as a continuous variable, and thus rendering it most suitable for the analysis [9]. Clustering was performed on each dataset separately, and the most consistent clusters were identified with the significant overlapping genes between the two datasets. After merging the clusters with redundant trends and exclusion of the clusters with *N* = 1, in both datasets two major age-associated expression profiles with opposite inclinations were identified (Figure 1A).

**Table 2:** Trends of the significant probes

Dataset	Positive regulation	Negative regulation	Total
**RS**	546	477	1023
**SHIP-TREND**	464	526	990
**Overlap**	210	165	378

Table shows the number of filtered (FDR < 0.05 and absolute FC ≥ 1.2) significant age-associated probes per trend of the expression profile, as well as the total for each of the datasets. The overlap of significant probes between the two datasets is also shown. The total overlap is higher than the sum of the overlapping probes, which present a positive regulation and of the probes presenting a negative regulation, as some probes can present a positive regulation in one of the datasets and a negative regulation in the other.

**Figure 2:**
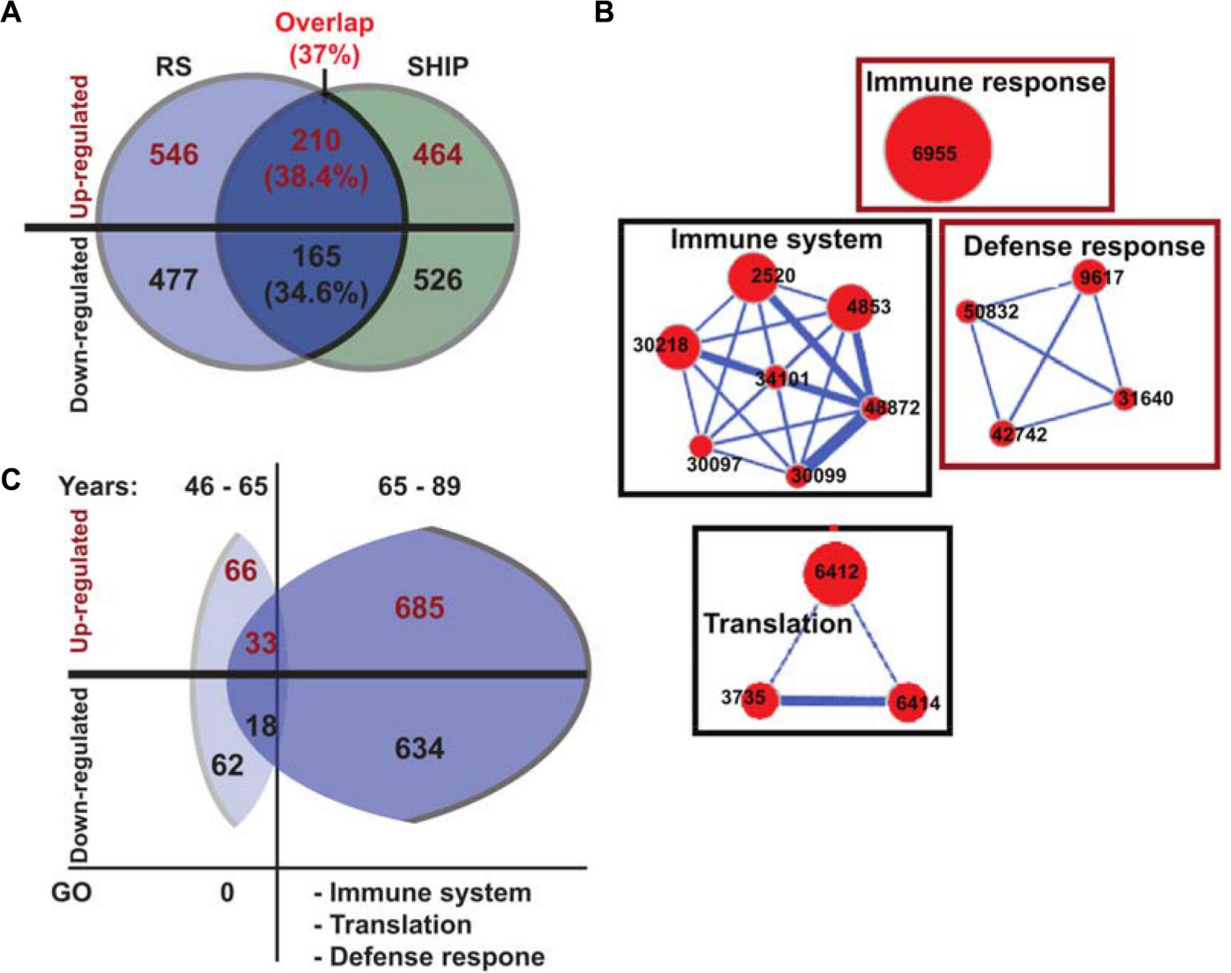
Gene network of the overlapping significant genes (**A**) Venn diagram showing the overlap of the significant (FDR < 0.05 and FC ≥ 1.2) age-associated probes in RS (blue) and SHIP-TREND (green) datasets. Up- or down-regulated genes are depicted in red or black text colour, respectively. In *parentheses,* the probe overlap in percentages is indicated, out of the RS dataset. (**B**) Cytoscape enrichment maps of enriched Gene Ontology (GO) groups in the overlapping genes from RS and SHIP-TREND. GO groups are denoted with the ID number of each term, and the size of the nodes is proportional to the number of genes associated to the node. Gene networks are connected with blue lines, a thicker line representing a stronger connection. Down regulated gene modules are gated black and the up regulated are gated red. (**C**) A schematic representation of the dysregulated genes in datasets subdivided for 46–65 years and 65–89 years. Numbers in *parentheses* show the percentages from the overlapping genes (in A), and indicates up or down regulated genes in each age group. Gene network clusters for each age group are specified.

To assess the age point at which the major changes in the expression profiles occur, *K*-means clustering using absolute correlation as a distance measure was applied. The age when major changes occur (i.e. age-position) was determined from the conjunction point between the opposite inclinations (Figure 1B). Importantly, only one age-position was identified in either dataset. In the RS dataset, the age-position occurred at the end of the seventh decade and in the SHIP-TREND dataset in the beginning of the seventh decade (Figure 2B). The earlier occurrence of the age-position in SHIP-TREND compared with RS could be attributed to the difference in the age ranges of the datasets. In a previous study, we showed that the age-position is influenced by the age range of the dataset [9]. Therefore, we then verified the age-position in matched age range (46 to 81 years of age) datasets. The whole analysis was repeated in the age-matched datasets, and 296 probes were filtered as significant in the RS, and 857 probes in the SHIP-TREND. Also in these datasets only one age-position was found, and it was mapped to the seventh decade in both RS and SHIP-TREND datasets (Supplementary Table S3 and Figure S3). This indicates that the difference in the occurrence of the age-position between the full and the age-matched datasets is due to the different age range covered by the two studies. Moreover, in the RS dataset only 21% of the genes that passed the *p-value* and fold change filters were found in the age-matched dataset. This suggests that most gene expression changes are denoted by the age group > 81 years old.

Subsequently, major age-associated molecular signatures in both datasets were identified using clustering of the gene functional groups into enrichment maps. Overall, enrichment maps in both datasets were highly similar, and the most prominent functional gene networks overlapped between the RS and the SHIP-TREND. Reduced expression levels were found for genes of RNA metabolism and translation groups, and an increase in expression levels was found for genes of the defense response and erythrocyte system (Supplementary Figure S4). Gene networks of the immune system were dysregulated without a specific dysregulation direction (Supplementary Figure S4).

The most consistent genes and gene networks were then identified by overlapping the significant probes between the two initial datasets. In total 378 dysregulated genes overlapped between the two datasets and for 375 genes dysregulation direction was the same as in the parental dataset (Figure 2A). The overlapping probes were enriched in the immune and defense responses, and in the translation machinery groups (Figure 2B). The genes associated with the translation network showed an age-associated down-regulation, whereas genes associated with the immune system presented a higher expression level with age (Figure 2B).

Next, we verified the molecular changes during the age-position. The RS dataset was split into two subsets with the age of 65 years being selected as a cut-off point. At 65 years the age-position was found in the two age-matched datasets (Supplementary Table S2). The younger group (< 65 years) comprised 606 individuals, whilst the older group (≥ 65 years) included 156 individuals (Supplementary Table S4). In the younger group, only 128 probes were found to be significantly age-associated and those were not enriched in any functional group (Figure 2C). Those probes were not found among the overlapping genes between RS and SHIP-TREND datasets (Supplementary Table S5). This suggests that molecular changes in blood prior to 65 years are neither robust nor consistent. In contrast, in the older age group 1319 probes were age-associated (FDR < 5% and absolute FC ≥ 1.2) and those were mapped to the immune system, translation and the defense response functional groups (Figure 2C). 65% of those genes overlapped with the significant probes from the SHIP-TREND (Supplementary Table S5). This indicates that major expression profile alterations in blood occur from the seventh decade onwards. This procedure was conducted in the RS study only as less subjects of older age are found in the SHIP-TREND dataset.

## DISCUSSION

Aging-associated molecular changes are highly prominent and are widespread across the genome. However, their progression and the rate of change are poorly understood, in part because linear models are most often applied for age-associated datasets. Here, we applied a quadratic model on two population-based datasets and found that in whole blood major expression profile changes occur during the seventh decade. This age-position was verified and was found to be robust and consistent. This age-position is in agreement with a recent study showing that the number of immune cells and T-cell receptor reduces from the seventh decade onwards [17]. Our analyses revealed that the most consistently and prominently affected gene networks are of the immune system and of the translation machinery. Those molecular signatures were also found in the RS dataset using a linear regression model [18]. We show that genes of the translation machinery showed reduced expression levels with age, suggesting reduced protein translation during aging in blood. Reduced total protein expression during the seventh decade was reported from proteomic studies in blood [19]. Moreover, slower rate of protein synthesis during aging was also reported [20, 21]. This suggests that the age-associated expression profiles reflect aging-associated functional changes in blood.

Whether or not the rate of molecular aging is similar between tissues is poorly understood. In whole blood, we identified only a single age-position during the seventh decade. A single age-position was found in kidney cortex, also during the seventh decade [9]. However, in brain frontal cortex and in Vastus lateralis muscle two age-positions were identified, the first during the fifth decade and a second one during early eighth decade [9]. This suggests that in humans the age at which major molecular changes occur differs between tissues. This conclusion in agreement with physiological studies suggesting that the rate of age-associated tissue deterioration differs between tissues [8]. Moreover, the prominent aging-associated gene networks also differ between tissues: translation and the immune system gene networks from blood were not identified in brain cortex or skeletal muscles tissues [9]. An age-position could indicate an aging-associated disease risk for tissue-specific disorders and could be a consideration for treatments and interventions during aging.

This methodology holds several limitations. Despite the fact that it is able to overcome the differences between the dataset platforms and identify the age-positions at which major changes in expression profiles occur, we note that the results from a quadratic regression model can fluctuate due to variations in the age range covered by the dataset and the age-dependent distribution of the samples. This effect is acknowledged, explained and quantified in our previous study on two different datasets from brain frontal cortex and Vastus lateralis muscle [9].

In population-based studies, the subjects are often not selected or filtered. This can cause a bias in the results, if certain confounders are not evenly distributed across age. For example, subjects are likely to differ in health features, lifestyle and socioeconomic status. We did not identify major outliers, suggesting that socioeconomic differences may not have a major contribution to the transcriptome in whole blood. We did not find an uneven gender distribution across the age, in both datasets. This indicates that the age-associated pattern we report here would not be caused by differences in the gender distribution across age. However, molecular aging highly differs between genders [7]. How the age-position differs between genders should be addressed in future studies. We found that subject distribution is skewed in the old age, and applied sample weighting that compensates for the uneven sample distribution across age. With this procedure, two independent datasets can be compared [4]. Sample weighting resulted in an increased number of age-associated transcripts that pass the *p-value* threshold. However, the majority of transcripts had an absolute FC < 1.2. To increase selectivity, we applied a fold change filter, but a confident fold change threshold for aging studies should be determined in future studies using an independent procedure.

## MATERIAL AND METHODS

### Datasets

We performed a differential expression study in human peripheral blood samples from two cross-sectional datasets from two independent large prospective, population-based cohort studies. The Rotterdam Study (RS) [13, 22] consists of an ethnically homogenous group of 762 Caucasian subjects aged 46–89 years in the district of Rotterdam, the Netherlands. The SHIP-TREND (Study of Health in Pomerania) [14, 15] contains 991 individuals with available gene expression and phenotype data aged 21 to 81 years from the German region of West Pomerania. The initial analysis was carried out in the RS dataset and replication was performed in the SHIP-TREND dataset. RS participants (aged 46 years and older) were all examined in some detail at baseline: they were interviewed at home and then had an extensive set of examinations in a specially built research facility in the center of their district. These examinations were repeated every 3–4 years in characteristics that could change over time. The participants in RS are followed for a variety of diseases that are frequent in the elderly. The study has been approved by the Medical Ethics Committee of the Erasmus MC and by the Ministry of Health, Welfare and Sport of the Netherlands, implementing the Wet Bevolkingsonderzoek: ERGO (Population Studies Act: Rotterdam Study). All participants provided written informed consent to participate in the study and to obtain information from their treating physicians. Sample collection and data generation are detailed in [13]. The RS cohort III expression dataset is available at the GEO repository under the accession GSE33828.

The Study of Health in Pomerania (SHIP-TREND) was conducted between 2008 and 2012. The SHIP population-based epidemiological study aims at investigating the risk factors of common, population-relevant diseases. The study design and the sampling methods as well as genotyping and gene expression measurement and methods (Illumina HumanHT-12 v3 Expression Beadchips) have been described elsewhere [14, 15, 23]. The medical ethics committee of the University of Greifswald approved the study protocol, and oral and written informed consents were obtained from all study participants. The SHIP-TREND expression dataset is available at the GEO repository under the accession GSE36382: 991 samples are available for analysis.

Both gene expression datasets have been obtained using the Illumina HumanHT-12 Expression Beadchip, but on different microarray platforms: v3 for SHIP-TREND and v4 for the RS-III respectively. The dataset demographics of both studies are summarized in (Table 1).

### Pre-processing

The gene expression levels were quantile-normalized and log2-transformed. Subsequently, probe and sample means were centered to zero. In the available RS dataset, probes were pre-filtered and declared significantly expressed when the detection *p*-values calculated by Illumina's GenomeStudio were < 0.05 in more than 10% of all samples. For the SHIP-TREND dataset, there was no filtering of the samples prior to analyses, thus all probes were available for analysis (Table 1). No further correction for other covariates or diseases was performed on the datasets, as it is not the purpose of this study. In order to discard possible outliers, a principal component analysis (PCA) was performed on both datasets using the *R-base*packages of the statistical environment R (Supplementary Figure S1). Eventually, no samples were discarded. Both datasets equally passed all processing steps with the same parameter settings in the models involved, as described below.

### Sample weighting

Prior to the selection of the most significant age-associated probes, the samples were chronologically ordered. The RS dataset is skewed towards the elderly, thus the distribution of samples across age is uneven (Supplementary Figure S2). This is caused by the design of the RS cohort (RS-III is the youngest cohort of RS: most of the older individuals already participated in RS-I or RS-II). In order to compensate for this uneven distribution and reduce the likely influence upon the statistical tests, sample weighting [24] using a dynamic age window was applied using the following procedure: each sample was assigned to an age group that consisted of samples in the range of ± 5 years from the age represented by the current sample. The weight value for each sample was calculated as being inversely proportional to the number of samples in its age group, following the equation:

$$w_{i}=\frac{1}{N_{i}}x_{i}$$ (1)

where *xi* is a sample from the dataset and *Ni* represents the number of samples present in the age group of a sample:

$$N_{i}=\sum_{\left(j=1\right)}^{n}\left[s_{j}=x_{i}\pm 5\right]$$ (2)

with *sj* being the number of samples *xi* for subject *j* in its designated age group.

### Probe smoothing

In order to reduce the inter-individual variation and facilitate the identification of age associated expression trends, the data was smoothed probe-wise. To achieve this, a simple quadratic regression model was used. The motivation behind this choice and a detailed description of the model used can be found in [9]. The calculated weights for each sample represented a parameter in the regression model, following the equation:

$$E_{\text{ij}}=\alpha_{i}=+\beta_{i}(x_{j}w_{j})+\gamma {\left(x_{j}w_{j}\right)}^{2}+\varepsilon_{\text{ij}}$$ (3)

where *E_ij_* is the intensity of probe intensity i for subject *j*, *x_j_* the age of subject *j, w_j_* the weight for subject *j*, α_*i*_, β_*i*_, γ_*i*_ are probe-specific regression parameters, and ε_*ij*_ is the residual error.

### Probe filtering

**Figure 3:**
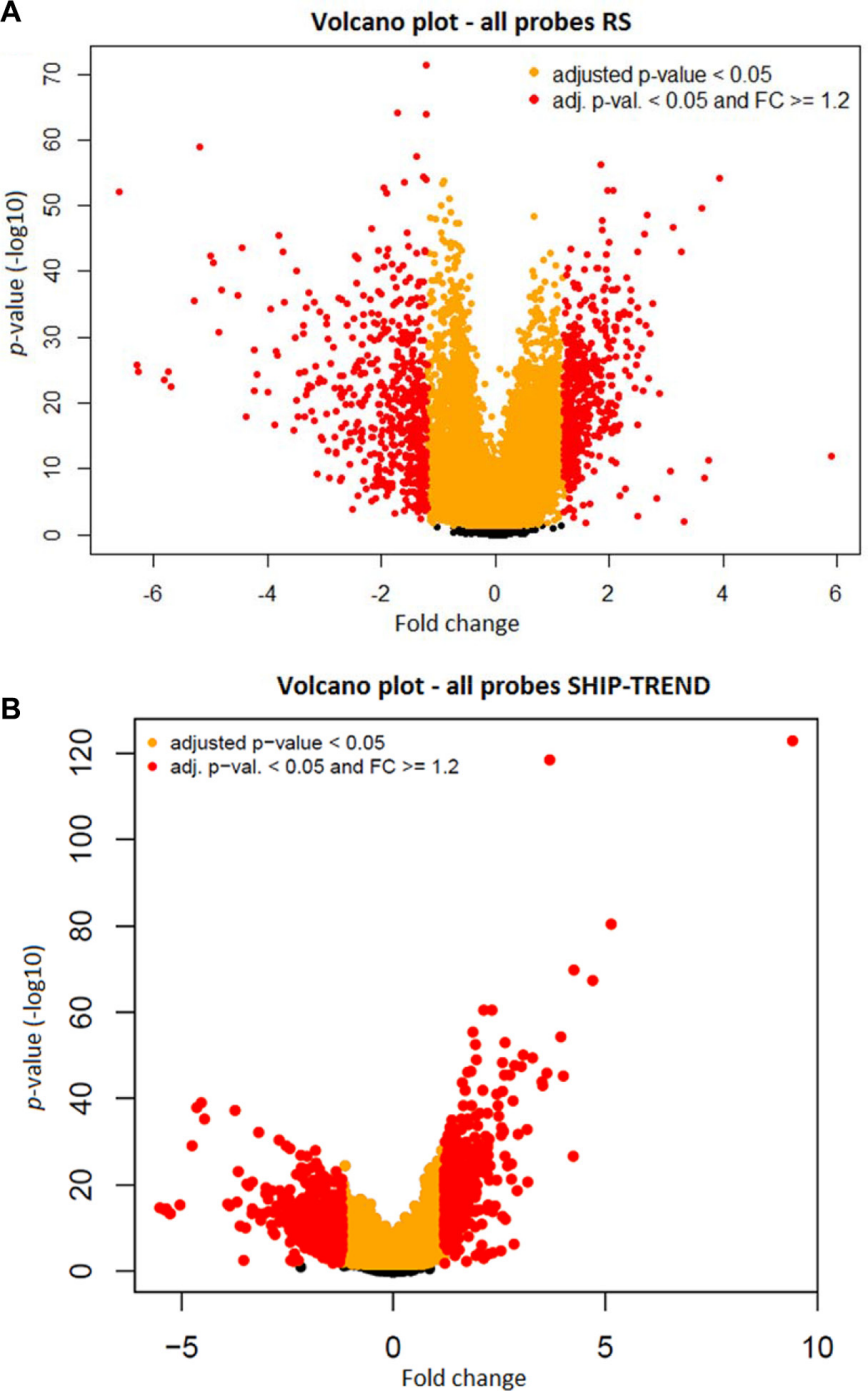
A volcano plot of all the probes from the RS dataset (**A**) and from the SHIP-TREND dataset (**B**):Volcano plots of the -log10 *p-value* (Y-axis) against the fold change (X-axis) show an association in the RS dataset (A) and in the SHIP-TREND dataset (B) In yellow, the probes with FDR < 0.5% and in red, the probes with FDR < 5% and an absolute fold change (FC) ≥ 1.2. In black are depicted the probes that did not pass the prior filters.

Filters were put in place in order to identify the significant age-associated probes. The *p-value* of each probe was calculated, testing the null hypothesis of no age association *versus* the quadratic regression model used in the prior step. The *p*-values were adjusted for multiple testing using the Benjamini-Hochberg false discovery rate (FDR) method. The probes presenting a FDR < 0.05 were considered statistically significant and kept for further analysis. Due to the large number of probes passing the *p*-value threshold (Figure 3 and Supplementary Table S2), a second filter set a threshold on the absolute value of the fold change (FC) [25]. There is no consensus on a FC threshold, but literature shows that an absolute FC ≥ 1.2 renders the results more likely to be reproducible [16]. Therefore, only probes that presented a FC ≥ 1.2 in absolute value were kept for the subsequent steps of the analysis.

### Clustering

The resulting set of significantly age-associated smoothed probes was standardized by subtracting the average intensity value of a probe from each of its data points. This allowed the probes to group based on the trend similarity rather than intensity. Grouping of the probes based on similarities in their expression profiles was achieved using *K*-means clustering as Euclidean distance measure of similarity. *K*-means clustering using absolute correlation as distance metric was used to identify the age-position. A detailed description of the clustering mechanism, as well as the statistical evaluation for confidence of the resulting clusters can be found in [9]. Major age-associated trends were identified after merging of the redundant clusters and exclusion of the single-gene clusters.

### Enrichment maps

The enrichment analysis was performed using the Entrez gene identifiers. The mapping of the probes to their Entrez gene identifiers was made with Illumina Human HT-12 v3 and v4 arrays platforms. From the significant probes, 2.8% were without annotation and were excluded. Enrichment maps were carried out using the entire set of probes as background in the DAVID 6.7 online analysis tool [26]. DAVID 6.7 analyses of the molecular functions and the biological processes of the gene ontology (GO) were performed. The analysis was carried out for each dataset separately, on the entire set of significant probes, as well as for the up or down regulated probes separately. Subsequently, the significant (*p* <0.05, FDR) GO terms were used as input in the Cytoscape 3.2.0 open source platform [27] and enrichment maps were created for up- and down-regulated genes separately.

### Supporting Data

The entire list of significantly age-associated genes for each dataset, as well as a list of the overlapping significant genes between the two datasets can be found in text format as supplementary material (Supplementary Tables S6–S8). These lists include the detected *p-value* after FDR, and the direction of regulation of each probe.

### Accession numbers

The accession number for the RS cohort III dataset used in this paper is GEO:GSE33828.

The accession number for the SHIP-TREND dataset used in this paper is GEO:GSE36382.

## SUPPLEMENTARY MATERIALS FIGURES AND TABLES








