# Supplementary material for: Blood RNA expression profiles undergo major changes during the seventh decade
Source: Oncotarget. 2016 Sep 17;7(44):71353–61. doi: 10.18632/oncotarget.12098 (PMC5342083; doi:10.18632/oncotarget.12098)
Supplement: Supplementary file 1 [file oncotarget-07-71353-s001.pdf]

Blood RNA expression profiles undergo major changes during the seventh decade

Supplementary Materials

Supplementary Table S1: Gender distribution across age in RS and SHIP-TREND datasets

Dataset	Variable	Min.	1st Qu.	Median	Mean	3rd Qu.	Max.
**SHIP-TREND**	Age distribution	20.0	40.0	50.0	50.1	61.0	81.0
	Female distribution with age	20.0	40.5	51.0	50.2	60.0	81.0
	Male distribution with age	22.0	39.0	50.0	50.0	61.0	80.0
**RS**	Age distribution	46.5	54.0	59.0	60.0	62.8	89.4
	Female distribution with age	47.3	54.1	59.4	60.4	63.5	89.4
	Male distribution with age	46.5	53.7	58.7	59.5	62.1	84.1

Gender distribution was assessed for the entire dataset with the samples chronologically ordered.

Supplementary Table S2: Comparisons of the sample-weighting and the age distribution on the number of significant probes and probe overlap

Study	#Probes with FDR < 0.05	#Probes with FDR < 0.05 and abs. FC ≥ 1.2	Probe overlap between weighted and non-weighted datasets (%)		
			All	Positive regulation	Negative regulation
RS	*4672 (22.%)*	*622*	76.7	74.2	80.2
	**15514 (73%)**	**1023 (6.6%)**			
SHIP-TREND	*4115 (8.4%)*	*574*	94.4	90.7	99.2
	**20373 (41.7%)**	**990 (4.8%)**			

In Italics, non-weighted datasets, in bold weighted datasets. In the second column, on the left side, between parentheses, the precentage of significant probes (FDR < 5%) from the total number of probes are depicted. On the right side of the second column, between parentheses, the precentages of probes passing FDR < 5% and presenting a FC ≥ 1.2 in absolute value are depicted, percentage calculated out of the significant probes (FDR < 5%). The last column depicts the precentage of probe overlap between non-weighted and weighted datasets per study. The overlap shows that the weighting of samples preserves the gene dysregulation in SHIP-TREND but less well in RS, due mainly to the difference in the sample distribution across age. In SHIP-TREND samples are more normally distributed, but in the RS the distribution is biased towards 50–60 years.

Supplementary Table S3: Number of significant probes found in the whole dataset and in the age-matched datasets as well as the identified age-position

Dataset	Sig. probes	Overlap	Age-position
RS all - 46-89	1023	378	69
SHIP-TREND all - 21-81	990		58
RS age matched - 46-81	296	106	63
SHIP-TREND age matched - 46-81	857		66

The upper part of the table contains the number of filtered significant probes (FDR < 0.05 and FC ≥ 1.2 in absolute value) from the entire dataset. The number of overlapping probes is denoted in the third column and the last column contains the age at which the identified age-position occurs. The lower part contains the number of filtered significant probes resulted from the subsets matched in age range (46 to 81 years of age), as well as their overlap, and the last column contains the occurrence point of the age-position.

Supplementary Table S4: Number of significant probes found in age-grouped (< or ≥ 65 yrs) subsets obtained from the RS dataset

Dataset	Nb. Samples	Up regulated	Down regulated	Total
**RS < 65**	606	66	62	128
**RS ≥ 65**	156	685	634	1319

The table shows the number of filtered significant probes (p-value < 0.05 after FDR and FC ≥ 1.2 in absolute value) by trend and in total, obtained from the two artificial datasets generated from the entire RS dataset divided at 65 years of age.

Supplementary Table S5: Overlap of the significant probes found in age-grouped (< or ≥ 65 yrs) subsets obtained from the RS dataset and the significant probes from entire RS and SHIP-TREND dataset

Dataset	FDR < 5%	Overlap RS (N = 1023)	Overlap SHIP-TREND (N = 990)	Overlap RS and SHIP-TREND (N = 378)
**RS < 65**	128	84 (65.62%)	57 (44.53%)	51 (13.49%)
**RS ≥ 65**	1319	654 (49.58%)	324 (24.56%)	272 (71.95%)

The table shows the number and percentage of overlapping probes (FDR < 5%) between the age groups formed from the RS dataset (less than 65 years of age and over 65 years of age) in the second column, and their overlap with: in the third column, the FDR < 5% probes obtained from all RS dataset; in the fourth column, the FDR < 5% probes obtained from the SHIP-TREND dataset; in the fifth column, the overlapping probes between the RS and SHIP-TREND datasets. The overlap is calculated out of the number of significant probes (FDR < 5%) obtained from the age groups (i.e., second column).

Supplementary Table S6: RS_all_significant_gene_list. See Supplementary_Table_S6

Supplementary Table S7: SHIP_all_significant_gene_list. See Supplementary_Table_S7

Supplementary Table S8: overlap_RS_SHIP_with_*p* values. See Supplementary_Table_S8

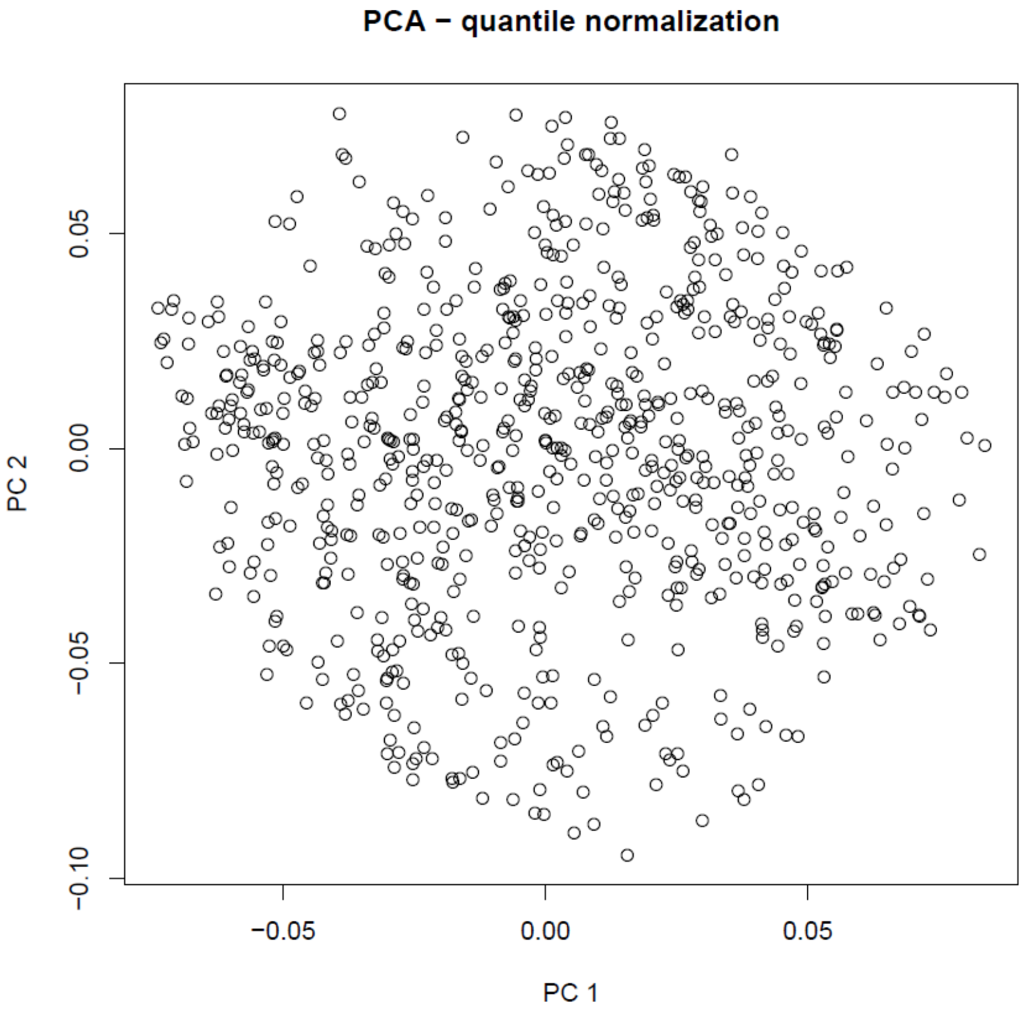

Supplementary Figure S1: The results of the principal component analysis (PCA) on the RS dataset, showing no outliers among the samples. The plot shows the first and second principal component plotted against each other.

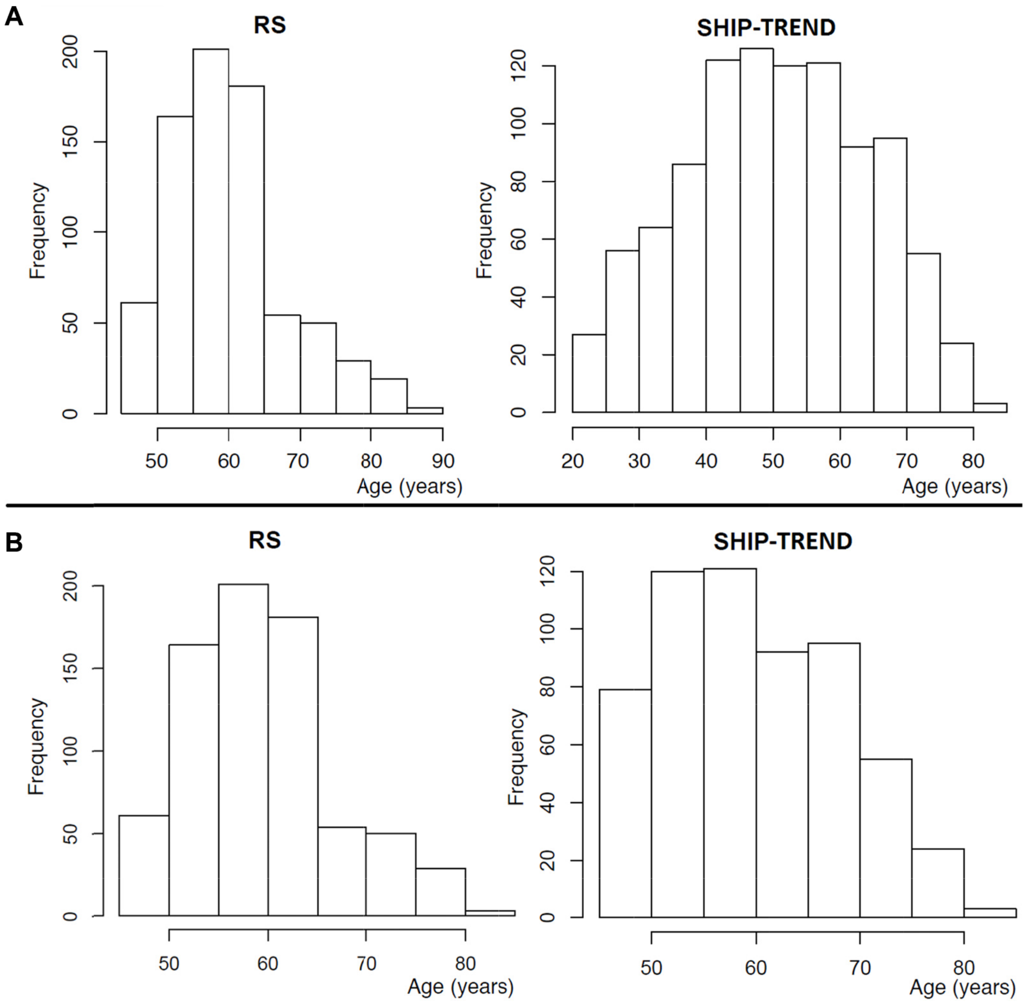

Supplementary Figure S2: Age distribution of the samples. Histograms showing the age distribution in the full RS and SHIP-TREND dataset (**A**) and in the artificial datasets with a matching age range from 46 to 81 years (**B**).

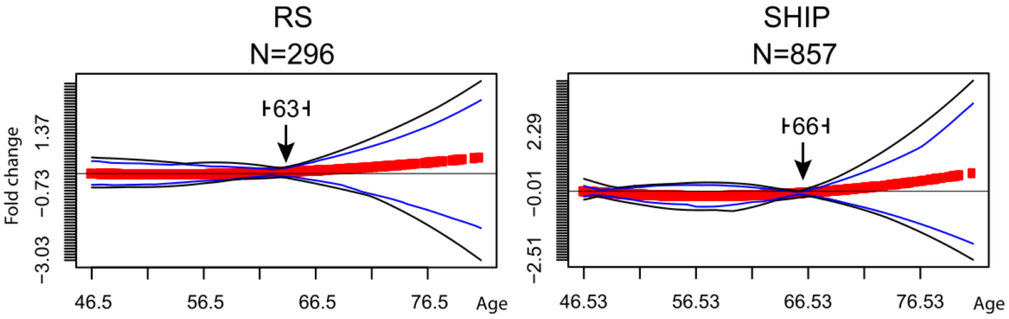

Supplementary Figure S3: The identification of the age-position in age-matched datasets. Plots show the absolute correlation clusters obtained from the age-matched datasets (46 to 81 years of age) generated from the RS and the SHIP-TREND dataset.

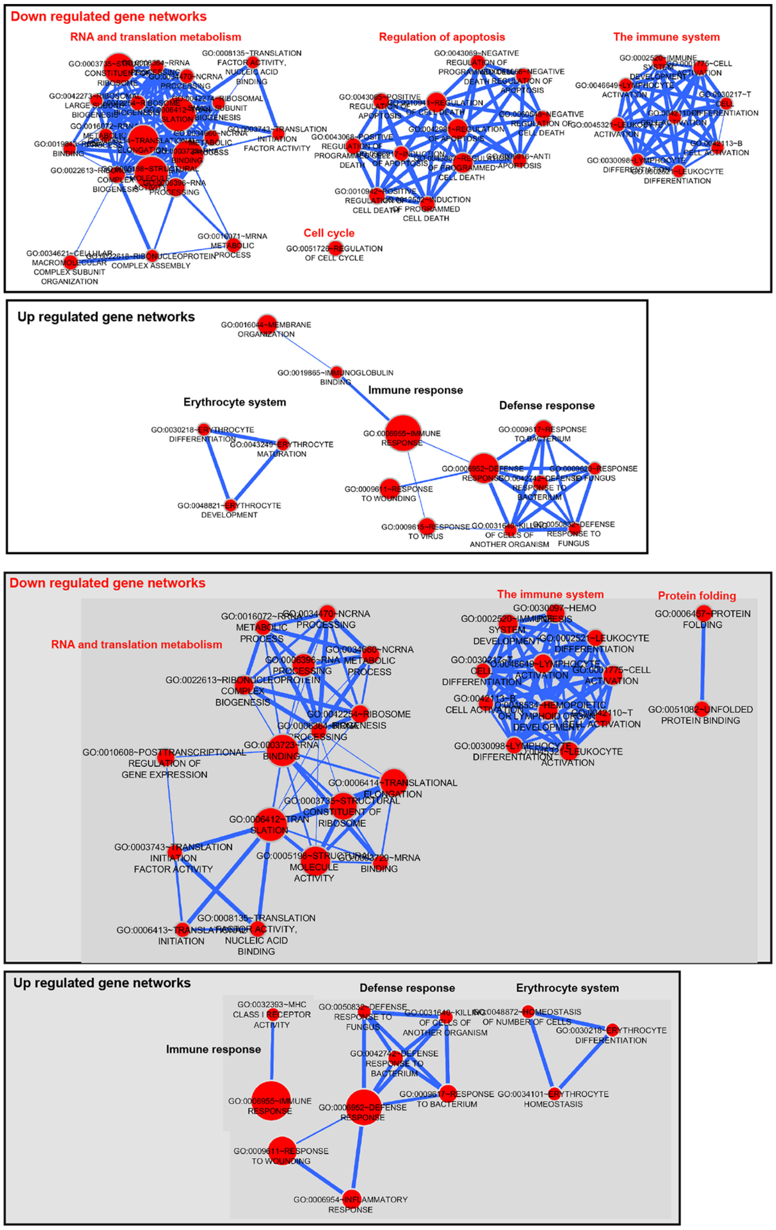

Supplementary Figure S4: Enrichment maps from the down- or up- regulated filtered age-associated genes in the RS (white boxes) or the SHIP-TREND (gray boxes) datasets. Enrichment maps of the filtered significant probes were generated using the open source platform called Cytoscape. Enrichment maps are contented with blue lines, a thicker line representing a stronger connection. The size of the nodes is proportional to the number of genes associated to the node.
